# Impact of Triton X-100 on Notch 1 Surface Receptor Immunofluorescence: A Cautionary Study

**DOI:** 10.15190/d.2025.5

**Published:** 2025-03-31

**Authors:** Maha Shahid, Kanwal Iftikhar, Shabana Usman Simjee

**Affiliations:** ^1^Hussain Ebrahim Jamal Research Institute of Chemistry, International Center for Chemical and Biological Sciences, University of Karachi, Karachi 75270, Pakistan; ^2^Dr. Panjwani Center for Molecular Medicine and Drug Research, International Center for Chemical and Biological Sciences, University of Karachi, Karachi 75270, Pakistan

**Keywords:** Cell permeability, Cortex, Immunocytochemistry, Notch 1 receptor, Primary culture, Triton X-100.

## Abstract

Triton X-100 is a cell-permeabilizing agent, widely used in molecular studies to enhance the permeability of cellular membranes. It has detrimental effects on the cell surface receptors thereby not suitable for surface receptor protein studies. Therefore, in the present study the protein expression of Notch surface receptor was analyzed with and without exposure of cell surfactant i.e. Triton X-100. It was observed that cultured cortical cells treated with Triton X-100 gave false protein expression due to the disruption of cellular membrane. On the contrary, cells without surfactant treatment exhibit fluorescence proportional to the true presence of Notch 1 receptors. This method can be followed for the analysis of any surface receptor protein through immunofluorescence to exclude any false expression.

## 1. INTRODUCTION

The Notch pathway is responsible for the maintenance of cell proliferation, cellular fate, differentiation and cell death. Itself, Notch is a receptor located on the cell surface and interact with transmembrane ligands like Delta (termed Delta-like in humans) and Serrate (termed Jagged in humans) transduces short-range signals on neighboring cells^1^. When ligand binds, two cleavage processes are initiated by Notch receptors, one is ADAM, which is disintegrin and metalloproteinase member, and the second is γ-secretase that is responsible for the release of Notch intracellular domain (NICD). NICD on translocating into the nucleus, forms a complex activated by co-activators and eventually activates target genes of Notch which are mammalian hairy and Enhancer of split (HES) and members of Hey family^2^.In biology, cell surfactants are widely utilized for extraction of proteins from cell membranes, protein crystallization, stabilization and denaturation and for enhancing the permeabilization of cell membranes. In immunocytochemistry, after the fixation of biological samples permeabilization has been done to access the intracellular antigens. Commonly used permeabilizing detergents are saponin, Triton X-100, and Tween-20. Saponin reacts with membranous cholesterols and leaves holes in the membrane by removing them. The other detergents like Tween-20 and Triton X-100 usually extract proteins and are non-selective in nature^3^. Triton X-100 is commonly used as nonionic surfactant for cell lysis and for the permeabilization of cell membrane. However, Triton X-100 have the ability to damage the cell surface receptors to enhance cellular permeability. Therefore, during imaging procedures for surface receptor proteins, the exposure to this surfactant causes disruption of receptors leading to false observations^4-7^.

Accordingly, we designed a comparative study to investigate the effect of Triton X-100 on immunofluorescence of Notch 1 receptor protein as summarized in schematic workflow. In one set of experiment Triton X-100 was used as a cell-permeabilizing agent and in the second set, cell permeabilization was not performed. These approaches were performed to evaluate the difference in Notch 1 protein expressions observed in both sets of experiments.

## 2. MATERIALS AND METHODS

### 2.1. Chemicals and Reagents

0.25% Trypsin-EDTA (Gibco, catalog # 25200056); Absolute Ethanol (MERCK, catalog # 64-17-5); Alexa Fluor 488 goat anti- rabbit IgG A11008 (Invitrogen, catalog # A11008); Amphotericin B (Biowest, catalog # L0009-050); DAPI (Life Technologies, catalog # R37606); Ethanol (RCI Labscan, catalog # AR1069-G2.5L); Fetal bovine serum (FBS) (Gibco, catalog # 10500064); Fluoromount-G Mounting Medium (Invitrogen, catalog # 00-4958-02); Formaldehyde 37% (SIGMA, catalog # 252549);Gibco Dulbecco's Modified Eagle Medium (DMEM) (Gibco, catalog # 11965092); ROTI®-ImmunoBlock (ROTH, catalog # T144.1); Notch 1 Antibody Polyclonal Antibody to Translocation Associated Notch Homolog 1 (TAN1) (Cloud clone, catalog # PAG797Mu01); PBS, pH 7.4 (Gibco, catalog # 10010031); Penicillin-Streptomycin (Gibco, catalog # 15140122); Poly-L-lysine 0.1% (Chem Cruz, catalog # Sc-286689)16.Sodium Pyruvate (Sigma-Aldrich, catalog # S8636); Triton® X-100 (Sigma-Aldrich, catalog # T8787); Trypan Blue Dye (MP Biomedicals, catalog # 195532).

### 2.2. Animal experiments

Neonatal rats (1-3 days old) of Sprague Dawley (SD) strain were provided by the Animal House Facility at the International Center for Chemical and Biological Sciences (ICCBS), University of Karachi. The use of protocol was permitted by the Scientific Advisory Committee on Animal Care, Use, and Standards, ICCBS (protocol #0012-2018). This study was conducted in accordance with the ethical guidelines established by above-mentioned committee.

### 2.3. Isolation of CTX cells

Primary cortical cultures were carried out as described previously with slight modifications^8^. The region of interest i.e., cortex was isolated from the rat pups under the laminar flow hood Class II, Type A2 Biohazard Safety Cabinet. The harvested tissue was washed with PBS followed by homogenization with pipetting in 1 mL fresh culture medium to form a fine cell suspension. After checking the count and viability, the cell suspension was transferred in culture flasks (25 cm^2^ and 75 cm^2^) containing high glucose DMEM and were then incubated at 37°C with 5% CO_2_. Cell morphology was examined daily, and medium was changed or added once or twice a week.

### 2.4. Passaging of cultured CTX cells

When cells achieved 75-80% confluency, medium from flask was removed, followed by washing with sterile PBS (pH 7.4) twice. After that, 0.05% trypsin-EDTA solution was added in a flask and incubated in incubator for 3-4 minutes. Once the cells were completely detached from the surface, fresh medium (twice the volume of trypsin-EDTA) was added immediately for neutralization purpose. Cell suspension was transferred in 15 mL falcon tube and centrifuged at 241 g for 5 minutes. After centrifugation, supernatant was discarded, and pellet was resuspended in fresh 1 mL culture medium and re-cultured in 25 cm^2^ or 75 cm^2^ flask.

### 2.5. Cell counting and cell viability using trypan blue exclusion test

To calculate the number of viable cells in cell suspension, Trypan Blue Exclusion Test was incorporated as reported previously. This test is based on the integrity of live cells to remains colorless (clear cytoplasm) by preventing the penetration of Trypan Blue, however dead cells fail to block its penetration and turns blue. Cell suspension was prepared by mixing with 0.4% trypan blue in a ratio of 1:1 or 1:2 in PBS (pH 7.4) and was loaded in both chambers of hemocytometer. The unstained (viable) cells were counted by viewing large corner square consisted of sixteen small squares at 10X magnification under the light microscope. The viable cells and their respective percentage were calculated by applying formulae mentioned below:

Number of viable cells/mL = Total number of viable cells counted x Trypan Blue Dilution Factor x 10,000

Percent viable cells = 100 x Number of cells excluding the dye / Total number of cells counted

### 2.6. Immunocytochemical Analysis of Primary Cultured CTX Cells

Immunocytochemical analysis was performed to evaluate the expression of Notch1 protein in primary cortical neuronal cultures. To culture cells, Poly-L-lysine (PLL) coating was performed under sterile conditions on round glass cover slips in 24-well plate. The cells were seeded at the density of 30,000 cells/well on cover slips. After that culture plate was incubated at 37ºC and 5% CO_2. _Once cells were confluent, they were fixed with 250 µL 4% paraformaldehyde fixative at room temperature for 15 minutes. After 15 minutes, fixative was removed, and cells were gently rinsed with 250 µL of 1X PBS. Cells were divided in two groups i.e. one group was treated with 250 µL 0.1% Triton X-100 and the other group was not treated with Triton X-100. Cells were incubated at room temperature for 10 minutes. This step is carried out to check the permeability of Notch 1 antibody with and without the presence of Triton X-100. After rinsing with PBS, 250 µL of ROTI^®^-ImmunoBlock was added and this time cells were incubated at 37ºC for 30 minutes. Then, blocking solution was removed and each group ofcells were treated with primary antibody Notch1 in a ratio of 1:600 (Table 1) and incubated at 37ºC for 1 h followed by overnight incubation at 4°C. Next day, cells were washed thrice with PBS followed by addition of 300 µL of secondary anti-goat antibody in each well in a ratio of 1:1000 (Table 1). Cells were re-incubated in dark at room temperature for 1 h. After 1 h, cells were washed and treated with DAPI (1 drop/mL) to counter stain nucleus for 5 min at room temperature. Following DAPI procedure, washing was done, and cover slips were mounted on glass slides using Fluoromount-G mounting medium.

**Table 1:** Table 1. Recipes of the solutions used in the study

S.No.	Solution	Reagent	Final concentration	Dilution vehicle	Storage Temperature	Shelf-Life
1.	1X Phosphate Buffer Saline (PBS)(pH 7.4)	PBS (10X)	1X	Deionized water	40C	1 month
2.	1X Blocking Solution i.e. ROTI®-ImmunoBlock	Blocking Solution(10X)	1X	1X PBS(pH 7.4)	Freshly prepared	-
3.	0.1% TRITON X-100 solution	TRITON X-100 (100%)	0.1%	1X PBS(pH 7.4)	Freshly prepared	-
4.	4% Paraformaldehyde solution	Formaldehyde (37%)	4%	1X PBS(pH 7.4)	Freshly prepared	-
5.	Primary Antibody Dilution	Notch 1 Antibody Polyclonal Antibody to Translocation Associated Notch Homolog 1 (TAN1)(1 mg/mL)	1.67 μg/mL	1X Blocking Solution	Freshly prepared	-
6.	Secondary Antibody Dilution	Alexa Fluor 488 goat anti- rabbit IgG(2 mg/mL)	2 μg/mL	1X PBS(pH 7.4)	Freshly prepared	-

### 2.7. Microscopic Analysis

Images from each slide were captured blindly using Nikon ECLIPSE Ni-E fluorescence microscope (Tokyo, Japan). The fluorescence for Notch 1 protein receptor and nucleus was analyzed using FITC and DAPI channel, respectively. After that, ImageJ2 software was used to subtract the background intensity of each image and for merging two images of Notch 1 and DAPI.

### 2.8. Statistical Analysis

The investigational data of the study is expressed as mean ± S.E.M. The significant mean differences between control and treatment groups were calculated by Statistical Package for the Social Sciences (SPSS) software (v 20). Data was evaluated using independent samples t-test. This test determines whether there is statistical evidence of a significant difference between the associated population means by comparing the means of two independent groups. Each experiment was performed five times (n=5) and each replicate was performed with similar experimental conditions. The significant levels indicated as *P ≤ 0.05, **P ≤ 0.01 and ***P ≤ 0.001.

## 3. RESULTS

### 3.1. Immunocytochemical analysis of Notch 1 marker in Triton X-100 treated and untreated groups

**Figure 1:**
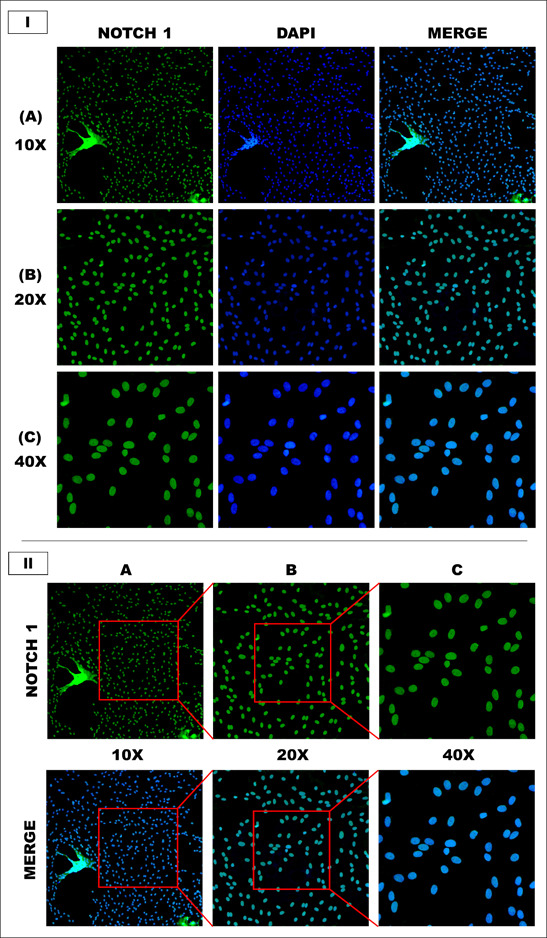
Figure 1. Immunofluorescence illustration of Notch 1 protein in primary cortical cultures treated with triton X- 100. (I) Panel A represents expression of Notch 1 protein with DAPI followed by merged image at 10X magnification. FITC indicates Notch 1 expression while blue color displays DAPI staining of the cell nuclei. Panel B and C displays the expression of Notch 1 protein with DAPI followed by merged image at 20X and 40X magnification respectively. The Notch 1 protein is expressed in almost all cells, which indicates that the receptor is damaged and displays the false results.(II) Panel A display Notch 1expression at 10X magnification, panel B display the zoomed region of panel A image at 20X. Finally, panel C shows zoomed region of panel B image at 40X magnification.

**Figure 2:**
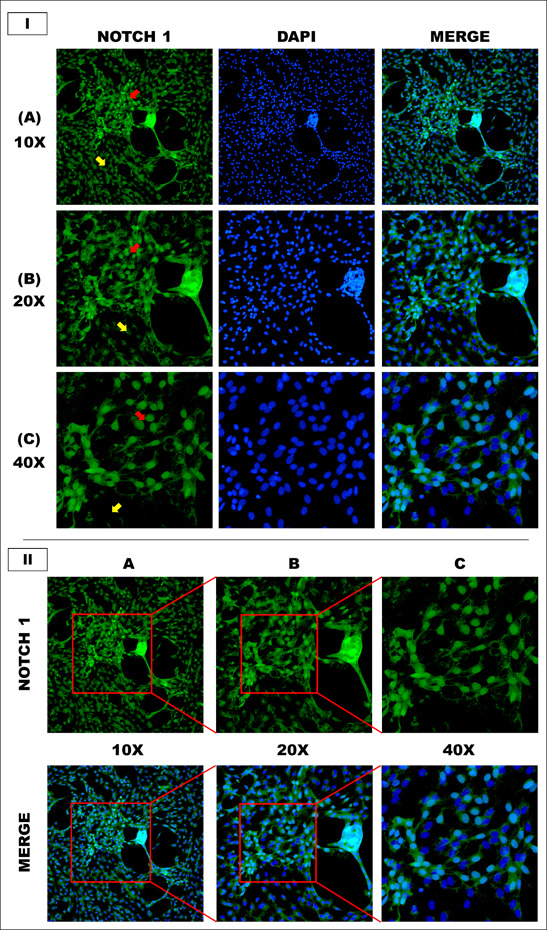
Figure 2. Qualitative Protein expression of Notch 1 in primary cortical cultures without treatment of triton X-100. (I) Panel A illustrates the Notch 1 protein expression at 10X magnification. FITC indicates Notch 1 expression while blue color displays DAPI staining of the cell nuclei. Panel B and C displays the expression of Notch 1 protein with DAPI and their respective merged images at 20X and 40X magnification. The red arrow points towards cells exhibiting Notch 1 expression while, Yellow arrow shows the cells with no Notch 1 expression. (II) Similarly, Panel A represents Notch 1 expression at 10X magnification, panel B demonstrates the focused region of panel A image at 20X. Lastly, panel C shows enlarged region of panel B image at 40X magnification.

The microscopic images of Triton X-100 treated group clearly shows that the Notch 1 expressing cells are same as the DAPI stained cells i.e. each cell is exhibiting the expression of Notch 1 protein (Figure 1). However, not every DAPI stained cell was expressing the Notch 1 protein in Triton X-100 untreated group (Figure 2). This observation indicated that Triton X-100 disrupt the receptor of Notch due to which the Notch 1 antibody accumulated intracellularly and gave false expression.

### 3.2. The validation of observed expression through statistical analysis

**Figure 3:**
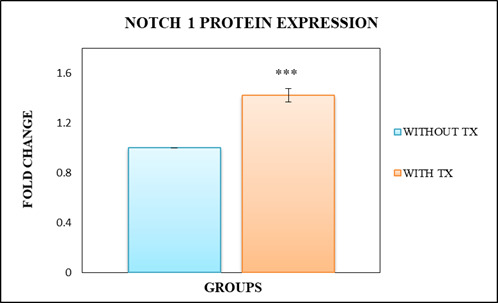
Figure 3. Graphical representation demonstrating the effect of Triton X-100 on the Notch 1 protein expression in the cortical cultured cells. Bar Graphs show fold change in Notch 1 expression in both Triton X-100 untreated and treated groups. The independent samples t-test was applied to examine the mean difference between both groups. Triton X-100 treated group established a significantly high Notch 1 expression (P ≤ 0.001) in comparison to the Triton X-100 untreated group. Each bar graph represents mean ± S.E.M of five individual experiments.

The statistical analysis of Triton X-100 treated group established a significantly high Notch 1 expression (P ≤ 0.001) in comparison to the Triton X-100 untreated group (Figure 3). This data was found to be the mirror image of the above statement.

## 5. DISCUSSIONS AND CONCLUSION

Cell-surface receptors are transmembrane proteins found in the membrane of target cells. These receptors have an extracellular domain that contains ligand-binding site, a transmembrane domain and an intracellular domain that initiates the signaling cascade inside the cells. Immunocytochemical analysis requires permeabilization of cell membrane for effective immunolocalization of target proteins^9^. Cell surfactants are found to be an effective tool for molecule delivery into the viable cells. One of them is Triton X-100, which is commonly used as nonionic surfactant for cell lysis and for enhancing the permeabilization of cell membrane^7,10^.In the present study, we have investigated the effects of Triton X-100 on protein expression of Notch 1 receptor. The protein expression of Notch 1 observed in non-treated group was much lower than that of Triton X-100 treated group. This indicated that Triton X-100 disrupt the receptor of Notch due to which the Notch 1 antibody accumulated intracellularly and gave false expression^11^. This protocol can be implicated to study the expression of the surface receptor proteins in immunofluorescence.It can be concluded that exposure of cell permeabilization agent i.e. Triton X-100 on cell surface receptor is detrimental in fluorescent microscopy. This was confirmed for Notch 1 receptor protein by fluorescence microscopy. The damage results in the false expression of Notch 1 protein when treated with surfactant. However, no such effect was observed on protein expression in Triton X-100 untreated group. This method can be applied for the identification of cell-surface proteins through immunocytochemical analysis.

## HIGHLIGHTS

- Triton X-100 is commonly used as nonionic surfactant for cell lysis and for the permeabilization of cell membrane.

- The exposure of Triton X-100 on cell surface receptor is detrimental in fluorescent microscopy.

- The damage results in the false expression of Notch 1 protein when treated with surfactant.
